# Supplementary material for: Five New Terpenes with Cytotoxic Activity from Pestalotiopsis sp
Source: Molecules. 2021 Nov 29;26(23):7229. doi: 10.3390/molecules26237229 (PMC8672272; doi:10.3390/molecules26237229)
Supplement: Supplementary file 1 [file molecules-26-07229-s001.zip › molecules-1446842-supplementary.pdf]

molecules

MDPI

Supplementary Materials

Five new Terpenes with Cytotoxic Activity from *Pestalotiopsis* sp.

Dan Zhao †, Meigeng Hu †, Guoxu Ma * and Xudong Xu *

Key Laboratory of Bioactive Substances and Resource Utilization of Chinese Herbal Medicine, Ministry of Education, Key Laboratory of Innovative Drug Discovery of Traditional Chinese Medicine (Natural Medicine) and Translational Medicine, Institute of Medicinal Plant Development, Peking Union Medical College and Chinese Academy of Medical Sciences, Beijing 100193, China; zhaodanonline@foxmail.com (D.Z.); mghu@implad.ac.cn (M.H.)

* Correspondence: mgxfl8785@163.com (G.M.); xdxu2012@163.com (X.X.); Tel.: +86-010-5783-3296 (G.M.)

† These authors contributed equally to this work.

Table of Contents Page

	Table of Contents	Page
**Figure S1**	^{1}H NMR Spectrum of **1** in DMSO-d_6 (600 MHz)	**1**
**Figure S2**	^{13}C NMR Spectrum of **1** in DMSO-d_6 (150 MHz)	**1**
**Figure S3**	HSQC Spectrum of **1** in DMSO- d_6	**2**
**Figure S4**	^{1}H-^{1}H COSY Spectrum of **1** in DMSO-d_6	**2**
**Figure S5**	HMBC Spectrum of **1** in DMSO-d_6	**3**
**Figure S6**	NOESY Spectrum of **1** in DMSO-d_6	**3**
**Figure S7**	ECD Spectrum of **1**	**4**
**Figure S8**	^{1}H NMR Spectrum of **2** in DMSO-d_6 (600 MHz)	**4**
**Figure S9**	^{13}C NMR Spectrum of **2** in DMSO-d_6 (150 MHz)	**5**
**Figure S10**	HSQC Spectrum of **2** in DMSO-d_6	**5**
**Figure S11**	^{1}H-^{1}H COSY Spectrum of **2** in DMSO-d_6	**6**
**Figure S12**	HMBC Spectrum of **2** in DMSO-d_6	**6**
**Figure S13**	NOESY Spectrum of **2** in DMSO-d_6	**7**
**Figure S14**	ECD Spectrum of **2**	**7**
**Figure S15**	^{1}H NMR Spectrum of **3** in DMSO-d_6 (600 MHz)	**8**
**Figure S16**	^{13}C NMR Spectrum of **3** in DMSO-d_6 (150 MHz)	**8**
**Figure S17**	HSQC Spectrum of **3** in DMSO-d_6	**9**
**Figure S18**	^{1}H-^{1}H COSY Spectrum of **3** in DMSO-d_6	**9**
**Figure S19**	HMBC Spectrum of **3** in DMSO-d_6	**10**
**Figure S20**	NOESY Spectrum of **3** in DMSO-d_6	**10**
**Figure S21**	ECD Spectrum of **3**	**11**
**Figure S22**	^{1}H NMR Spectrum of **4** in DMSO-d_6 (600 MHz)	**11**
**Figure S23**	^{13}C NMR Spectrum of **4** in DMSO-d_6 (150 MHz)	**12**
**Figure S24**	HSQC Spectrum of **4** in DMSO-d_6	**12**
**Figure S25**	^{1}H-^{1}H COSY Spectrum of **4** in DMSO-d_6	**13**
**Figure S26**	HMBC Spectrum of **4** in DMSO-d_6	**13**
**Figure S27**	NOESY Spectrum of **4** in DMSO-d_6	**14**
**Figure S28**	ECD Spectrum of **4**	**14**
**Figure S29**	^{1}H NMR Spectrum of **5** in DMSO-d_6 (600 MHz)	**15**
**Figure S30**	^{13}C NMR Spectrum of **5** in DMSO-d_6 (150 MHz)	**15**
**Figure S31**	HSQC Spectrum of **5** in DMSO-d_6	**16**
**Figure S32**	^{1}H-^{1}H COSY Spectrum of **5** in DMSO-d_6	**16**
**Figure S33**	HMBC Spectrum of **5** in DMSO-d_6	**17**

**Figure S34**	NOESY Spectrum of **5** in DMSO-d_6	**17**
**Figure S35**	ECD Spectrum of **5**	**18**
**Figure S36**	Details for ECD calculations of **1**	**18**

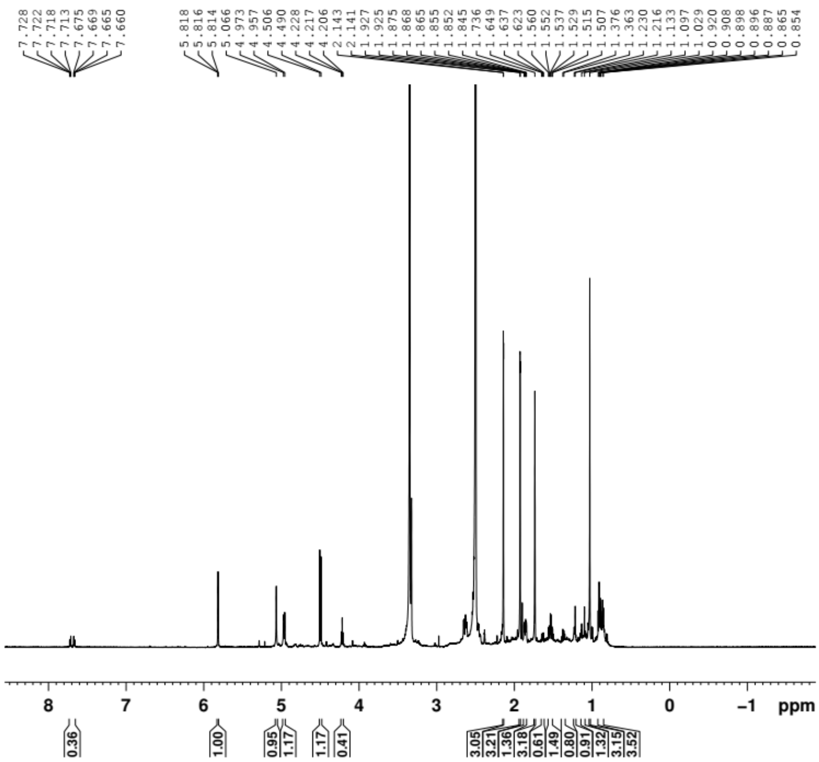

Figure S1. ^{1}H NMR Spectrum of **1** in DMSO-d_6 (600 MHz).

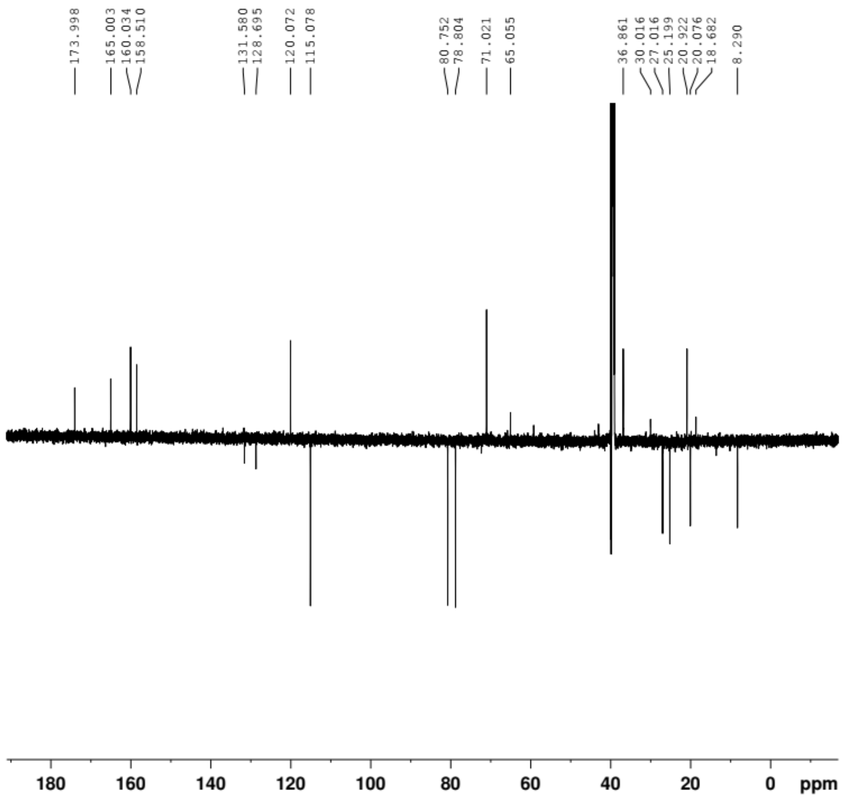

Figure S2. ^{13}C NMR Spectrum of **1** in DMSO-d_6 (150 MHz).

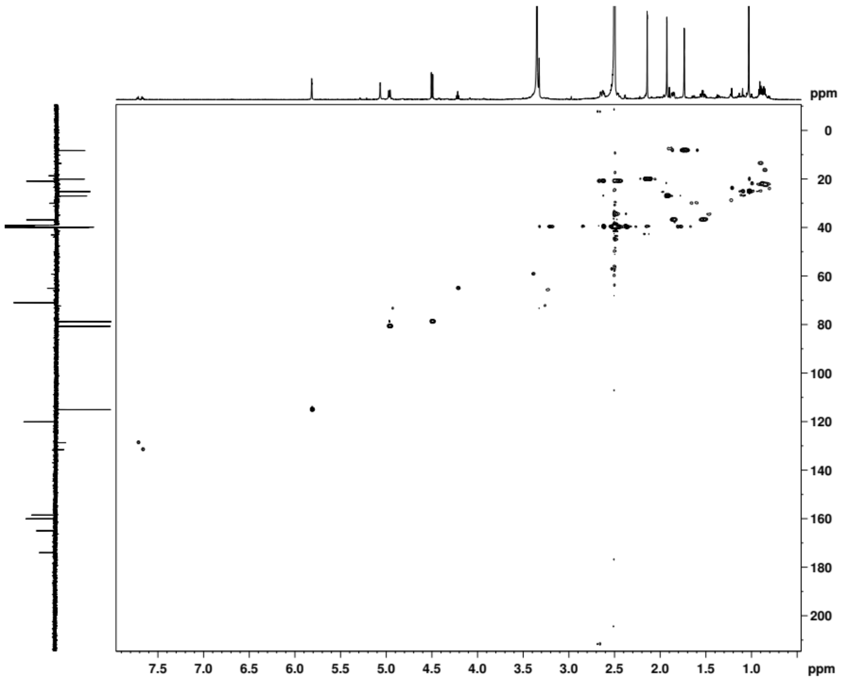

Figure S3. HSQC Spectrum of **1** in DMSO-d_6.

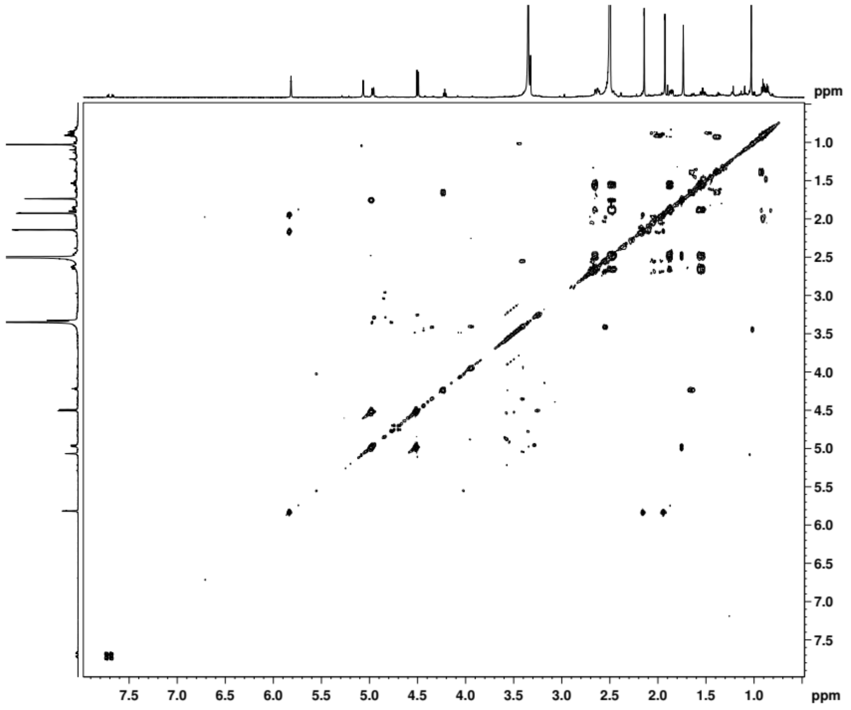

Figure S4. ^{1}H-^{1}H COSY Spectrum of **1** in DMSO-*d*$_6$.

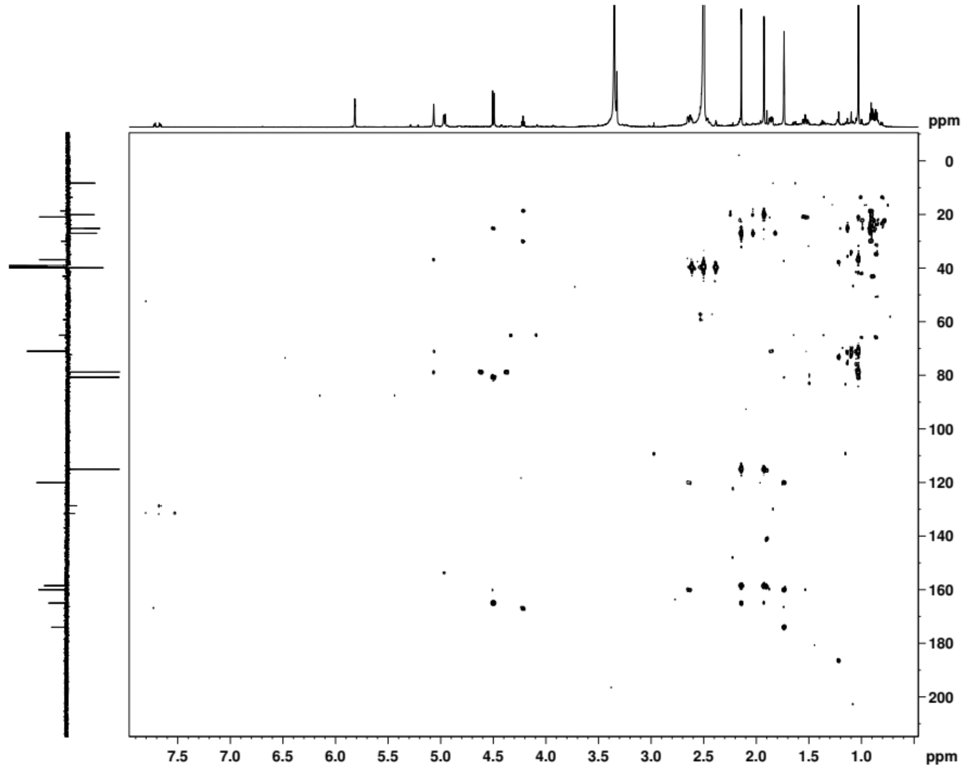

Figure S5. HMBC Spectrum of **1** in DMSO-*d*$_6$.

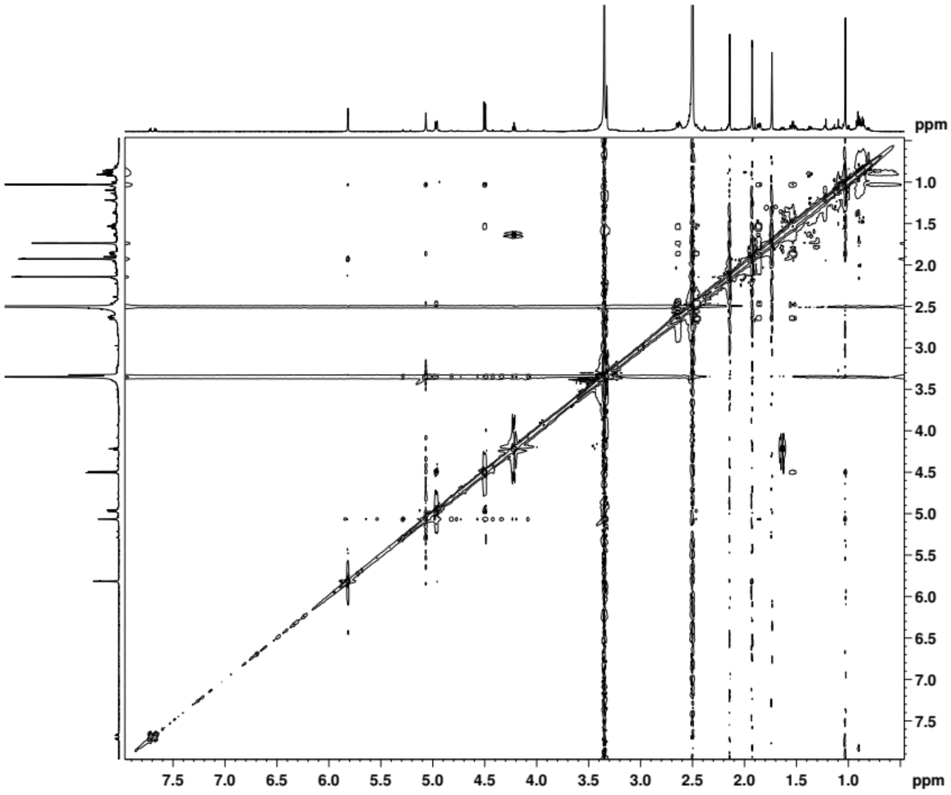

Figure S6. NOESY Spectrum of **1** in DMSO-*d*6.

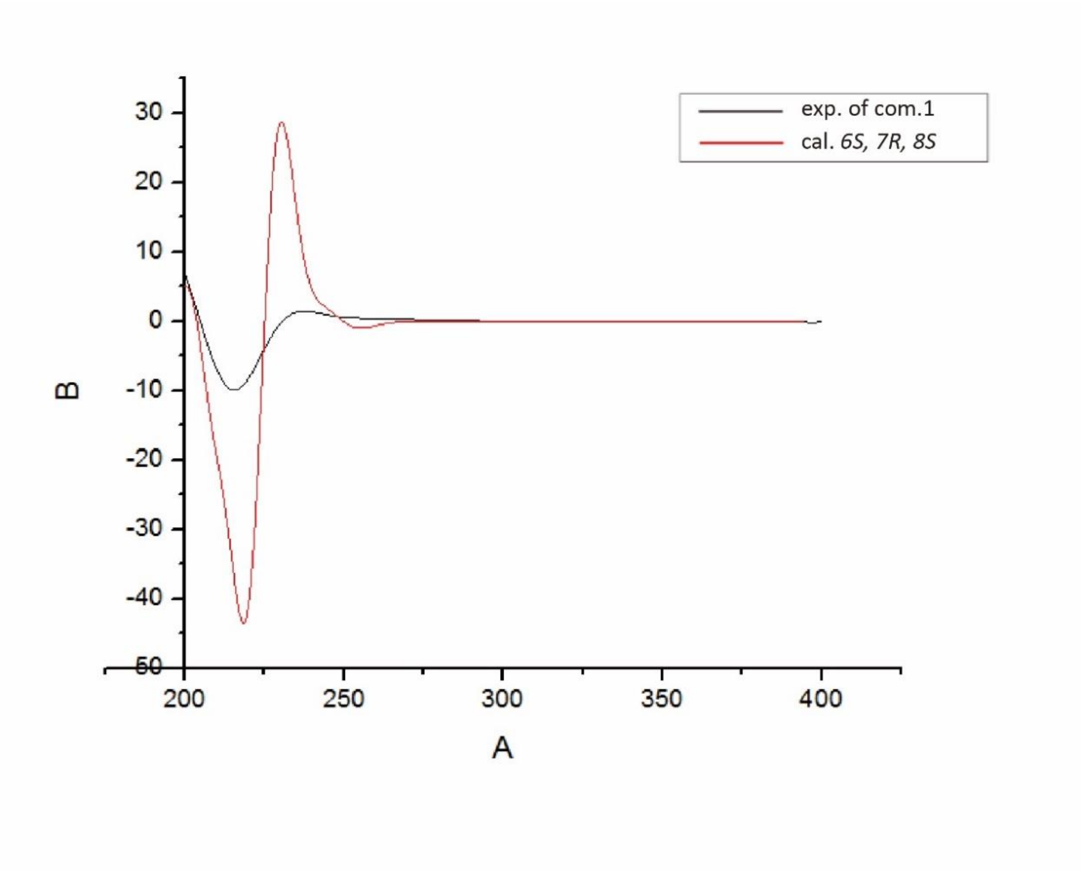

Figure S7. ECD Spectrum of **1.**

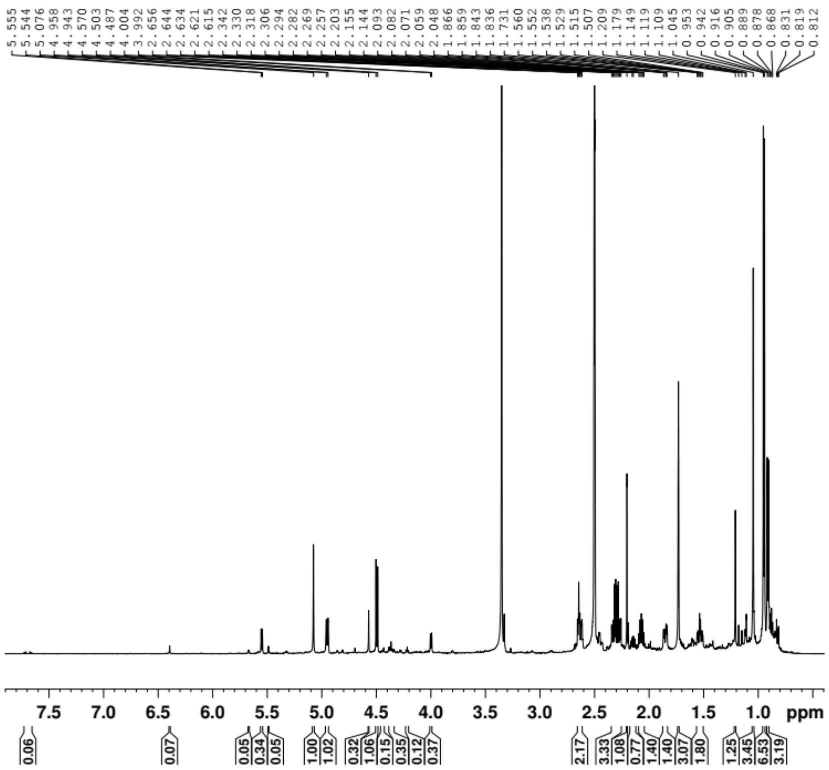

Figure S8. ^1H NMR Spectrum of **2** in DMSO-d_6 (600 MHz).

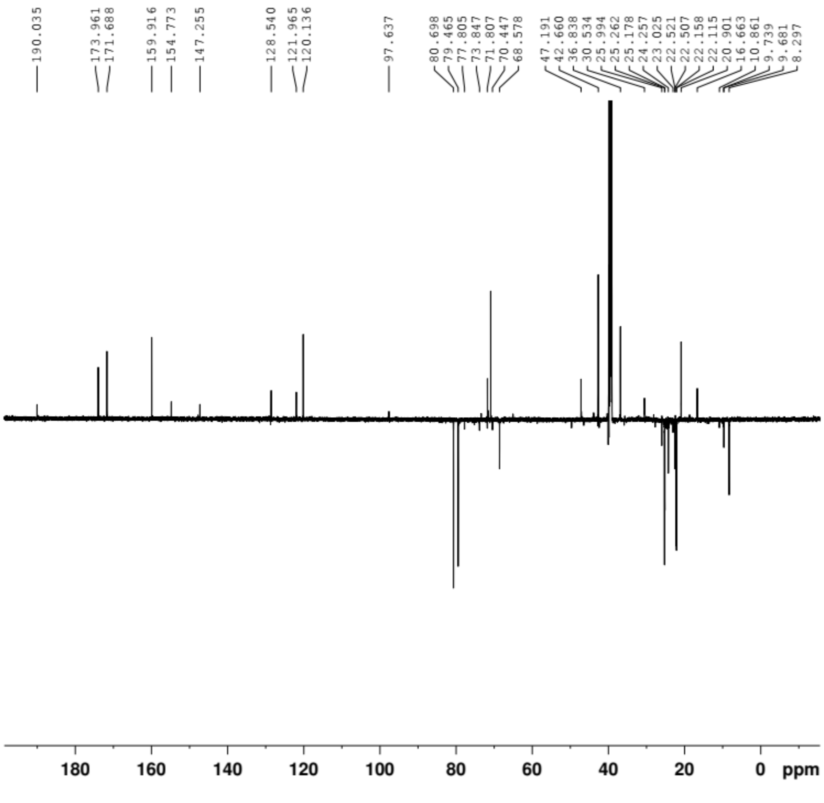

Figure S9. ^{13}C NMR Spectrum of **2** in DMSO-d_6 (150 MHz).

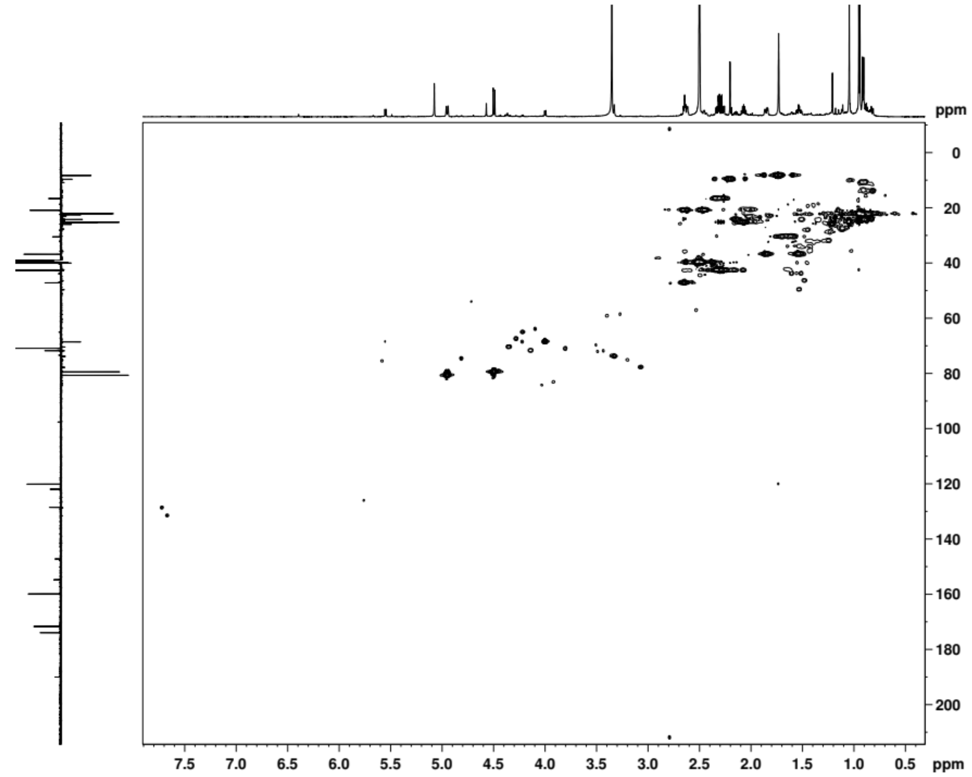

Figure S10. HSQC Spectrum of **2** in DMSO-*d*6.

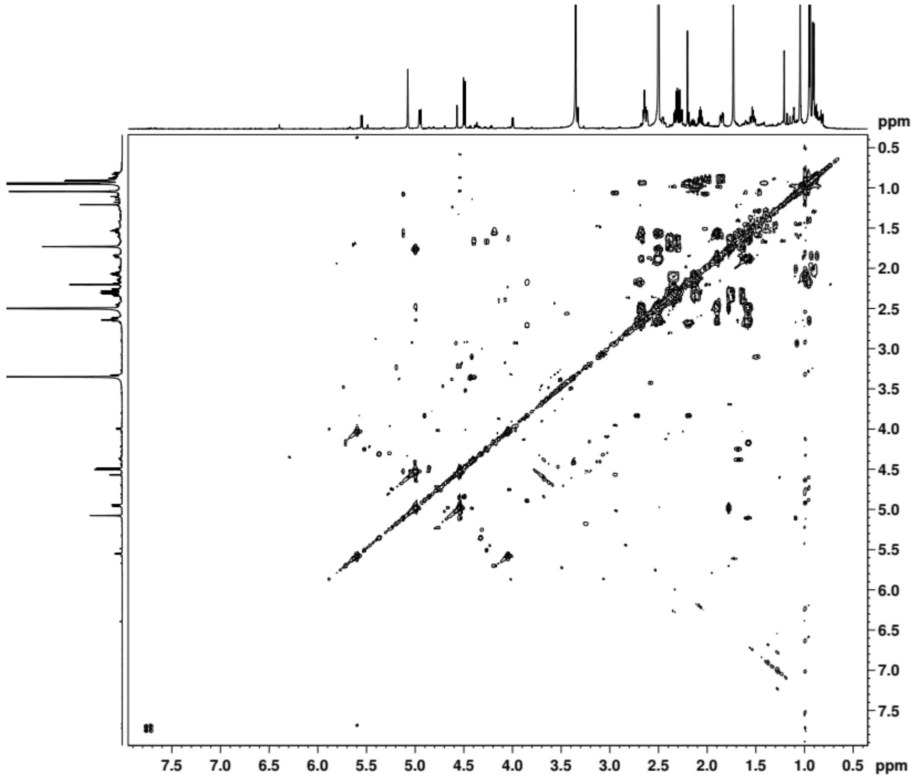

Figure S11. ^{1}H-^{1}H COSY Spectrum of **2** in DMSO-*d*6.

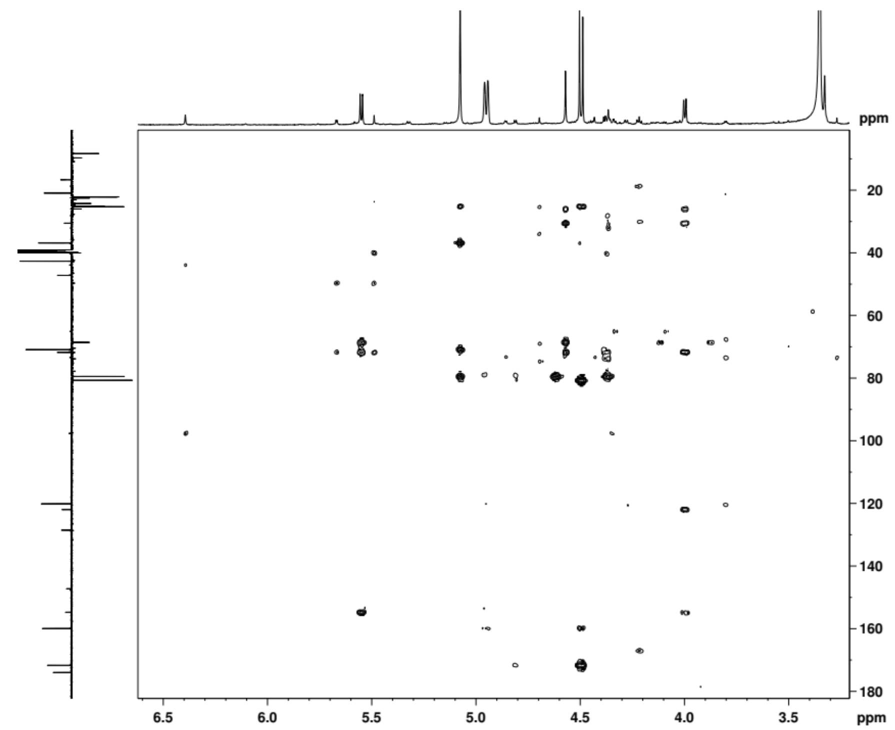

Figure S12. HMBC Spectrum of **2** in DMSO-*d*6.

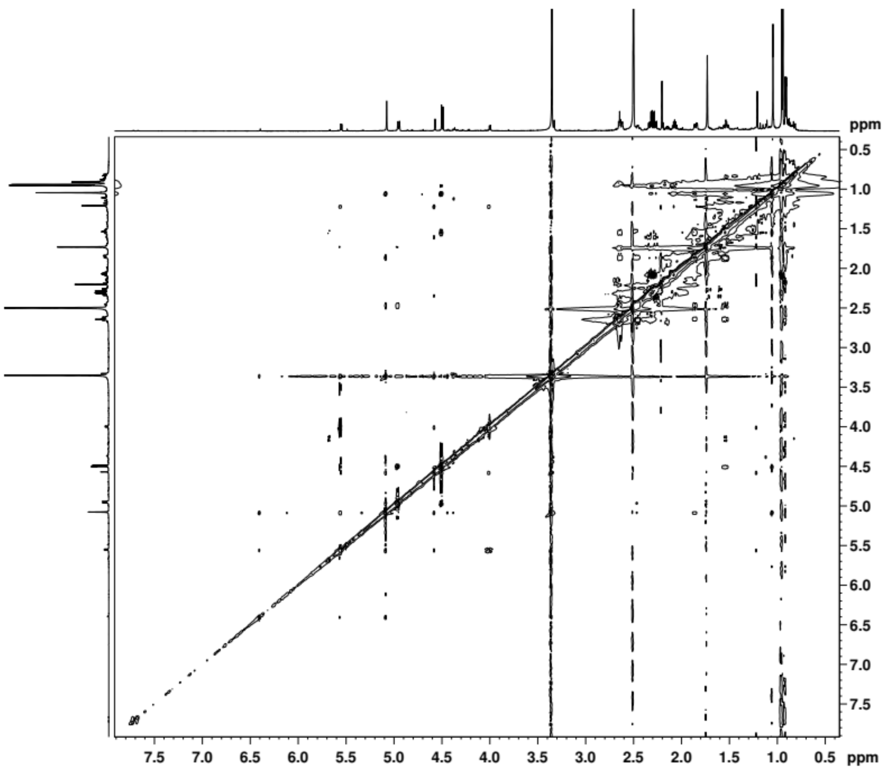

Figure S13. NOESY Spectrum of **2** in DMSO-*d*6.

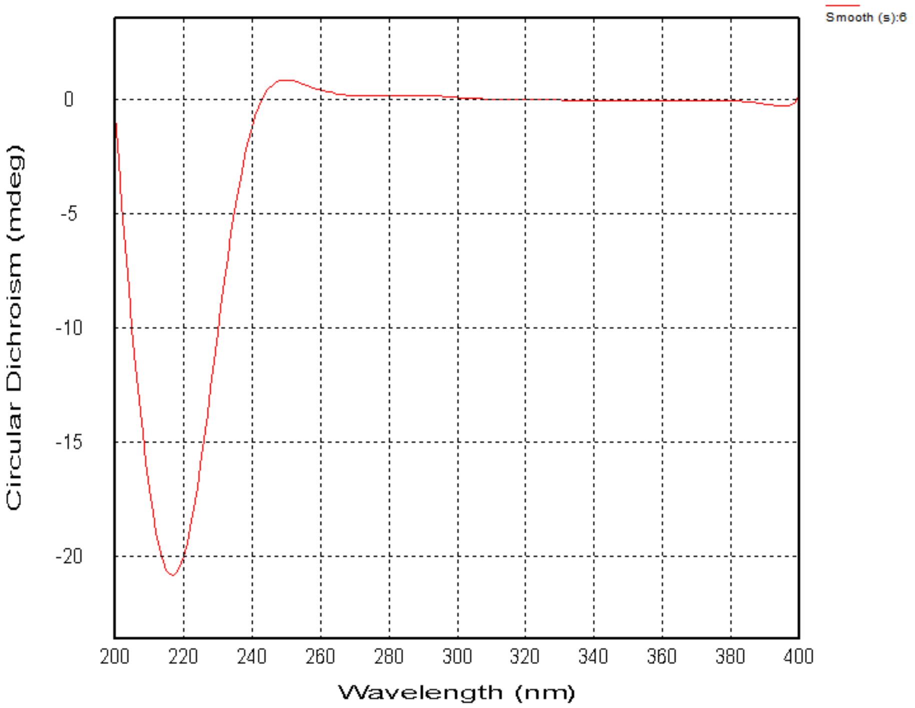

Figure S14. ECD Spectrum of **2.**

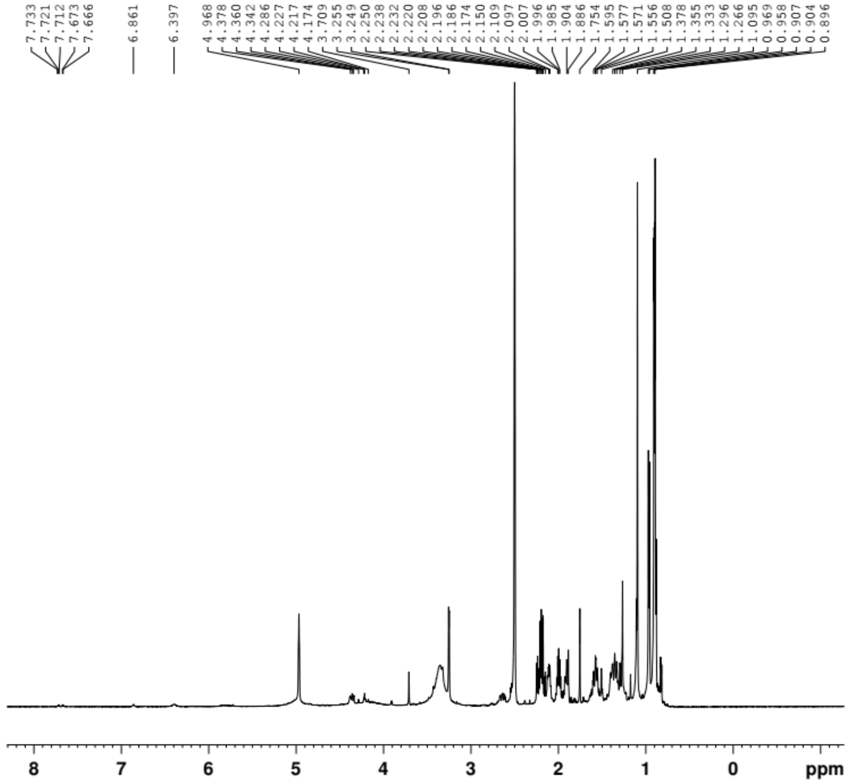

Figure S15. ^{1}H NMR Spectrum of **3** in DMSO-d_6 (600 MHz).

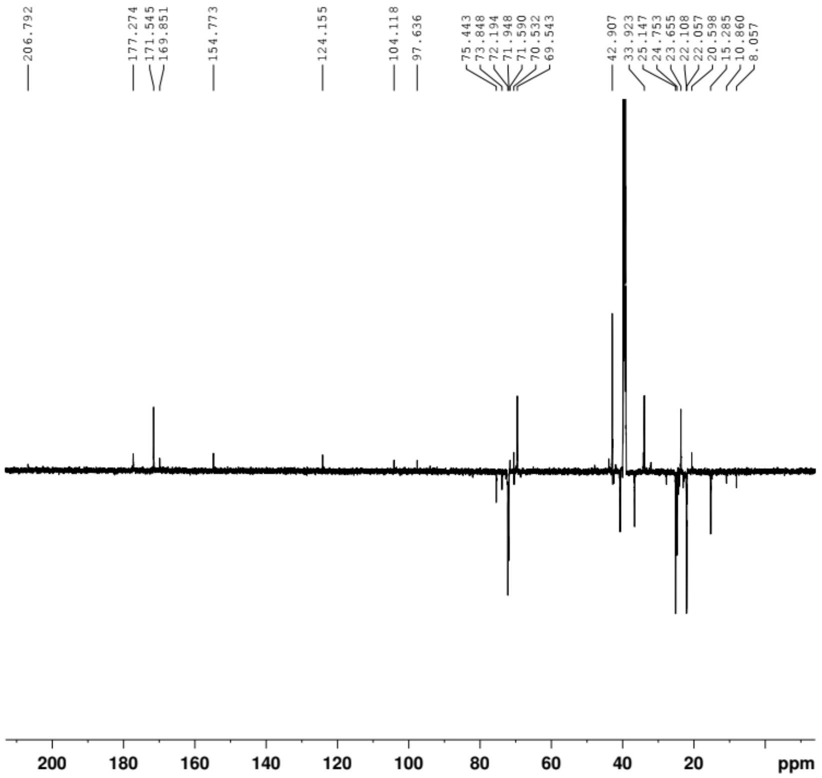

Figure S16. ^{13}C NMR Spectrum of **3** in DMSO-d_6 (150 MHz).

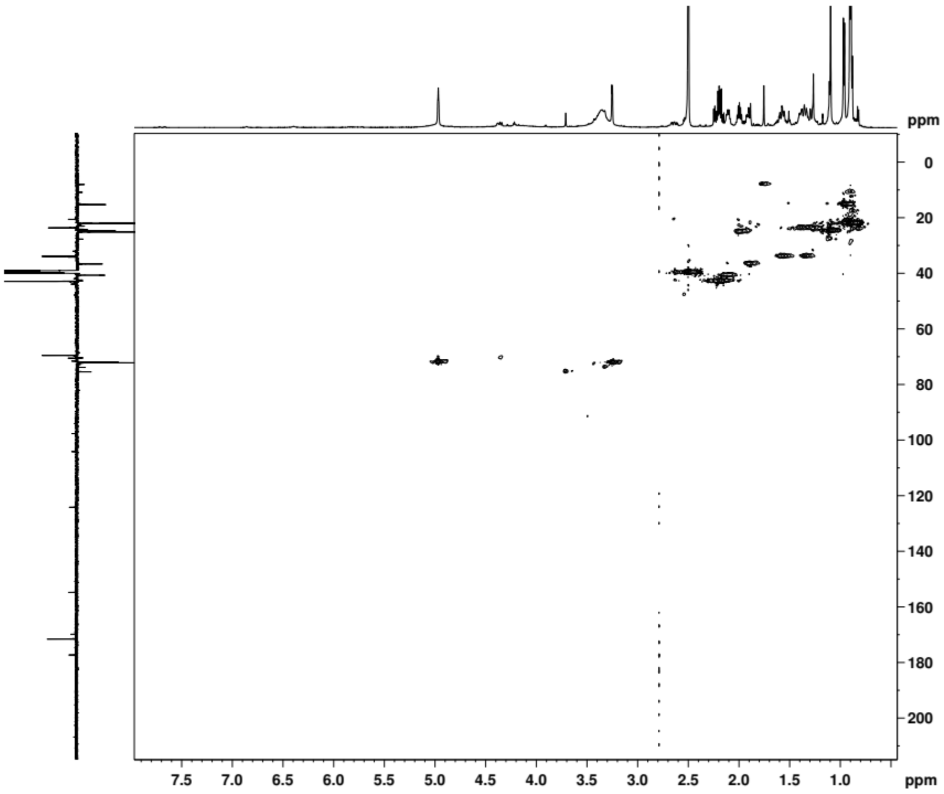

Figure S17. HSQC Spectrum of **3** in DMSO-d_6.

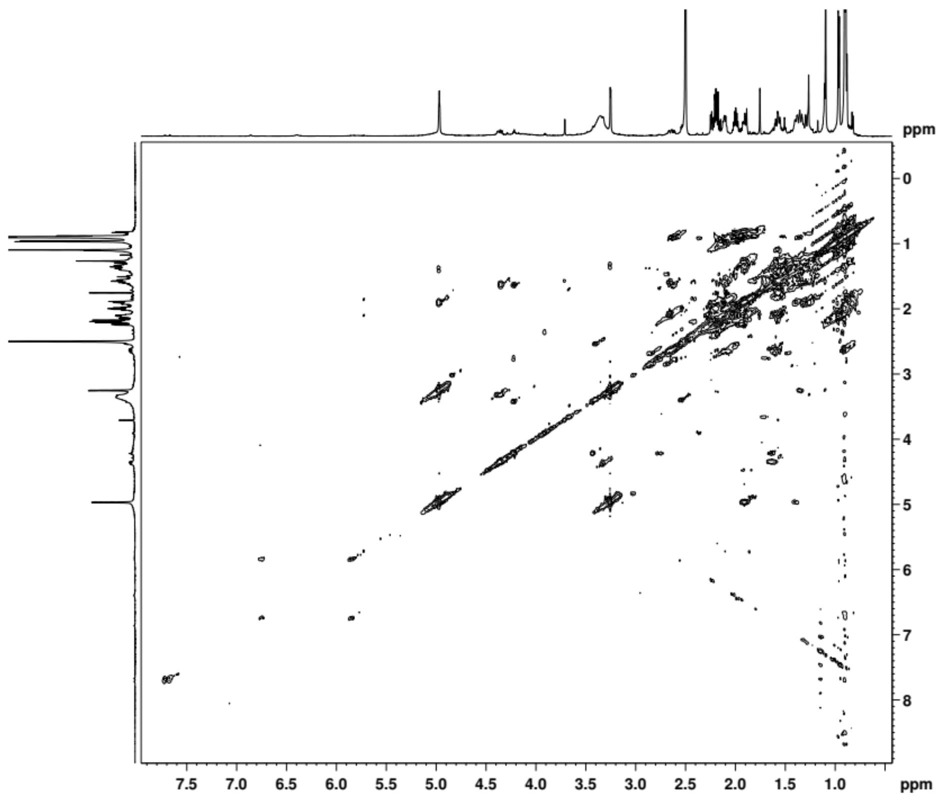

Figure S18. ^{1}H-^{1}H COSY Spectrum of **3** in DMSO-d_6.

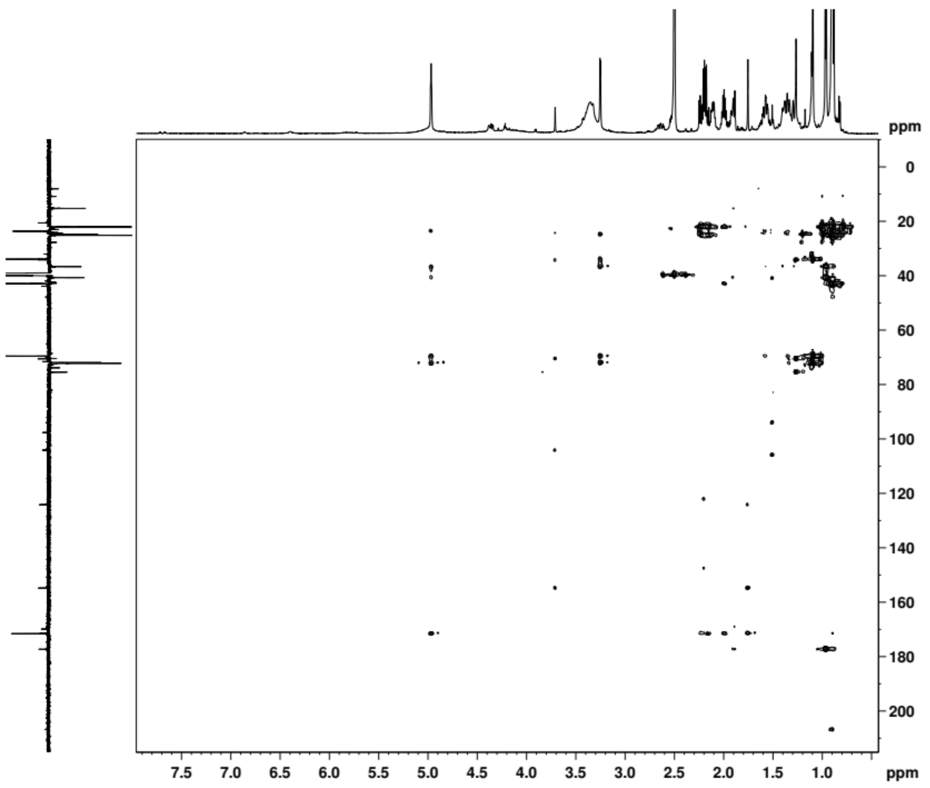

Figure S19. HMBC Spectrum of **3** in DMSO-d_6.

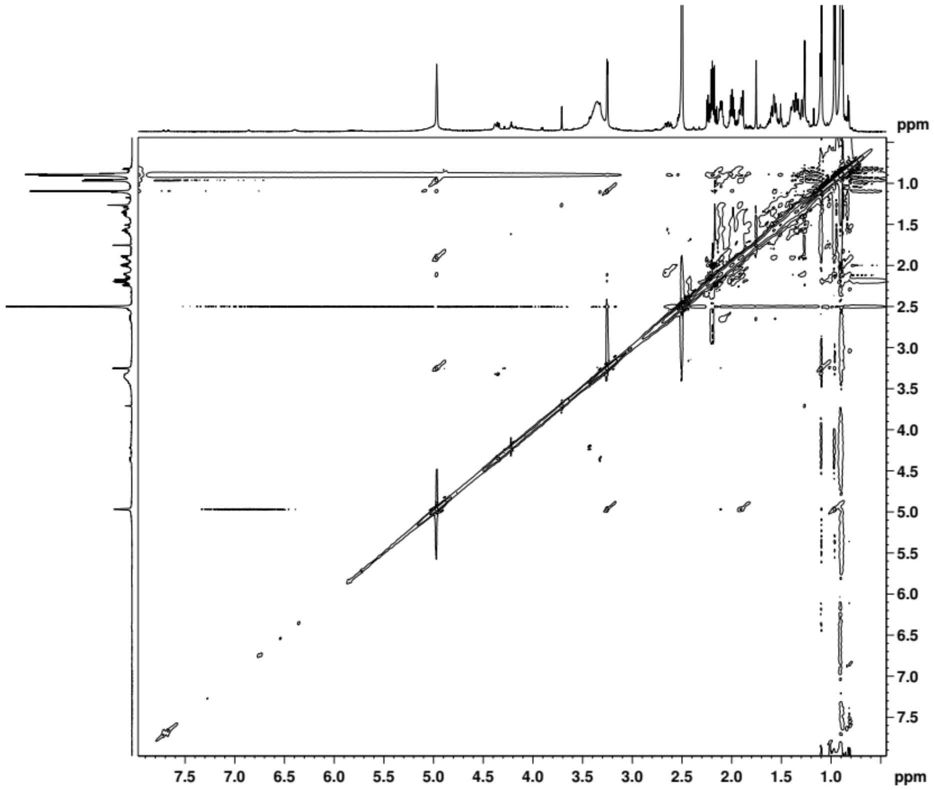

Figure S20. NOESY Spectrum of **3** in DMSO-d_6.

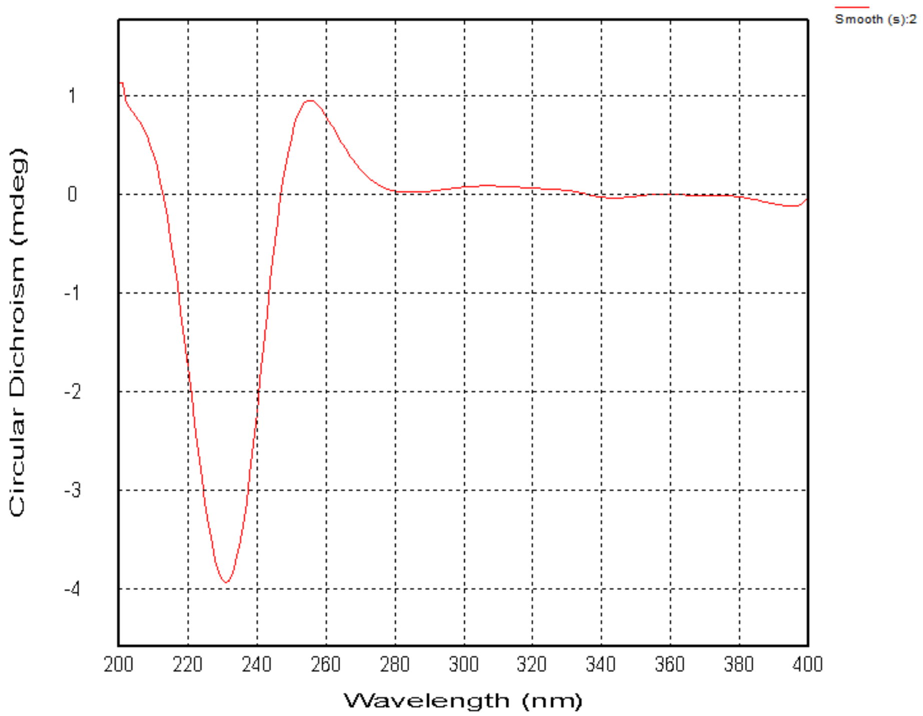

Figure S21. ECD Spectrum of **3.**

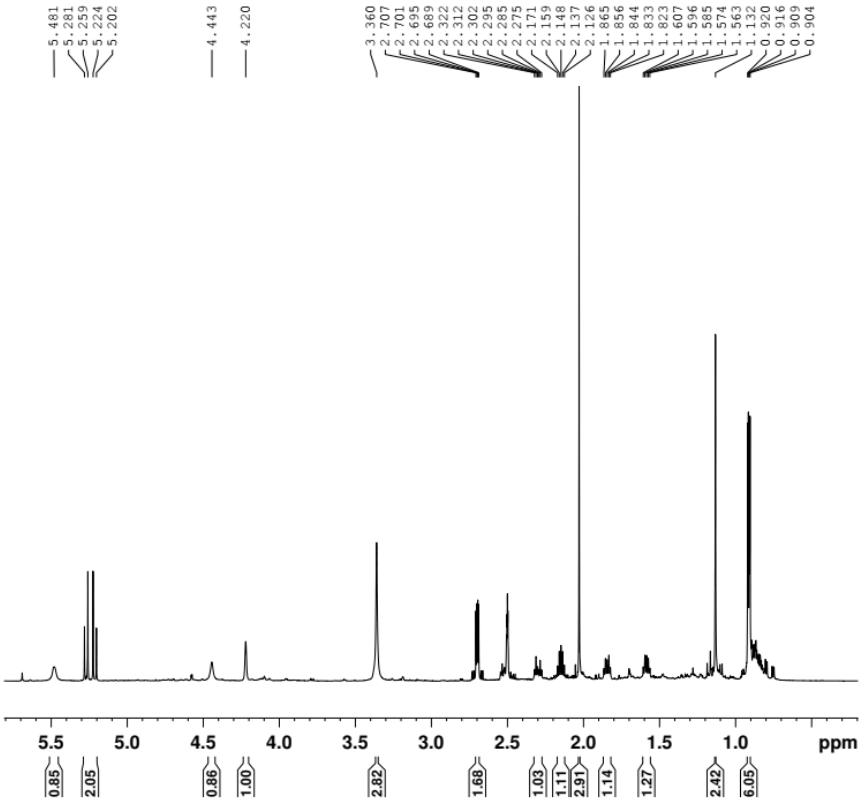

Figure S22. ^{1}H NMR Spectrum of **4** in DMSO-d_6 (600 MHz).

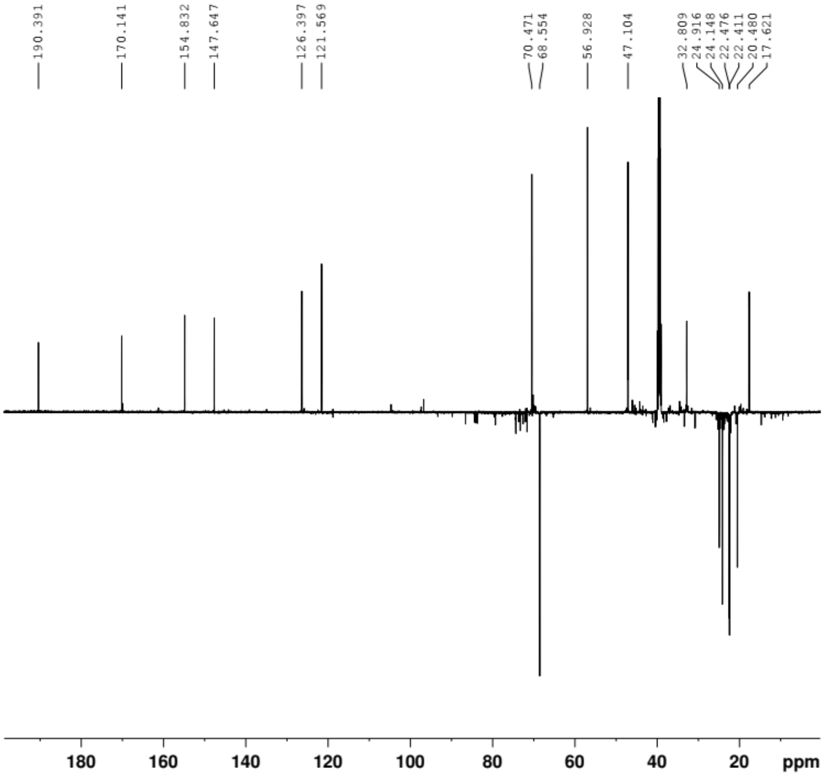

Figure S23. ^{13}C NMR Spectrum of **4** in DMSO-d_6 (150 MHz).

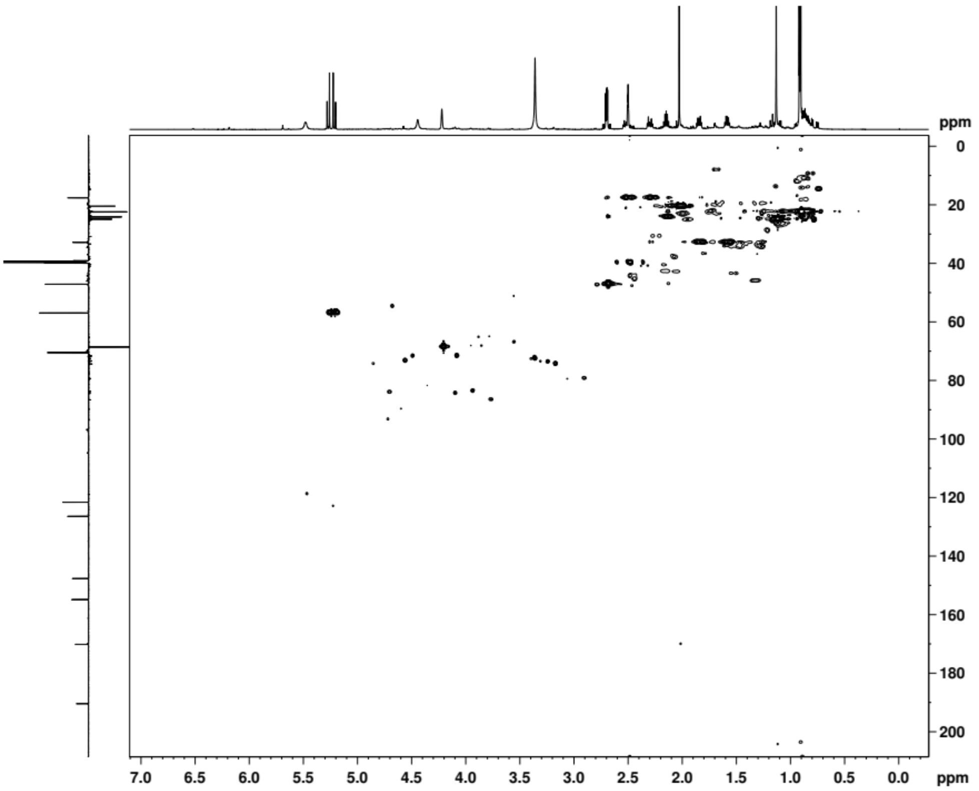

Figure S24. HSQC Spectrum of **4** in DMSO-d_6.

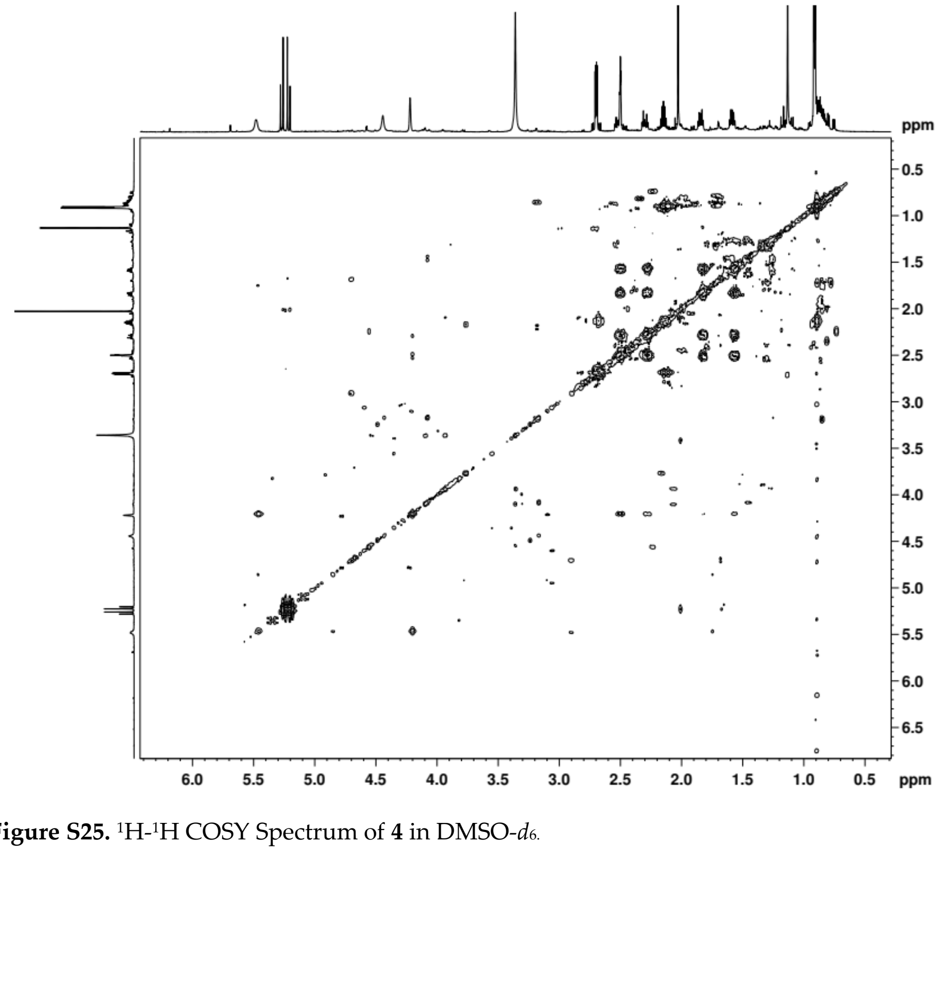

Figure S25. ^{1}H-^{1}H COSY Spectrum of **4** in DMSO-d_6.

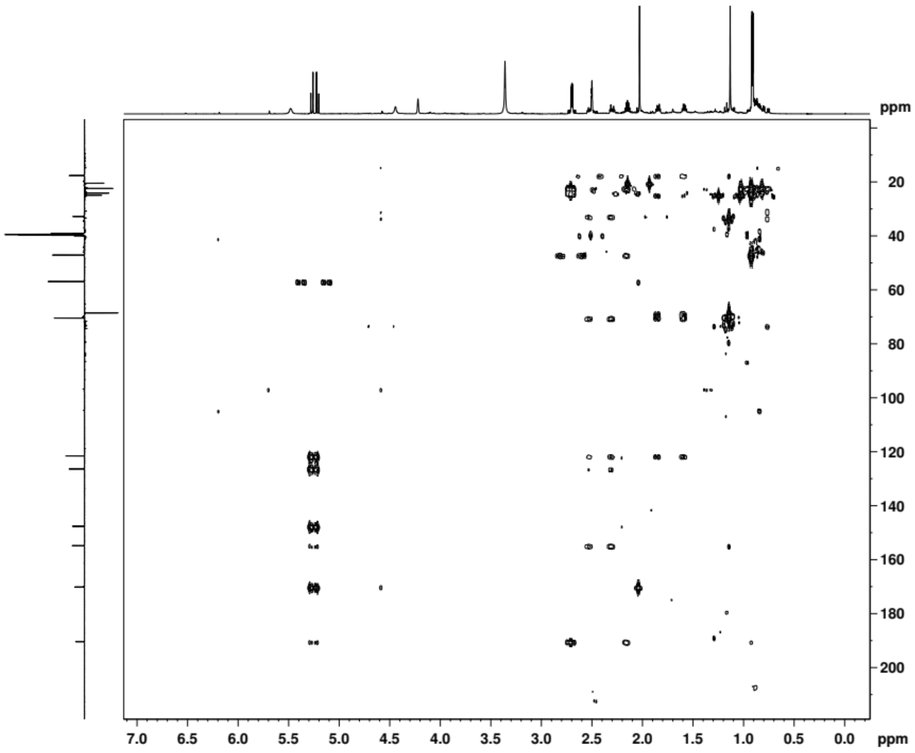

Figure S26. HMBC Spectrum of **4** in DMSO-*d*6.

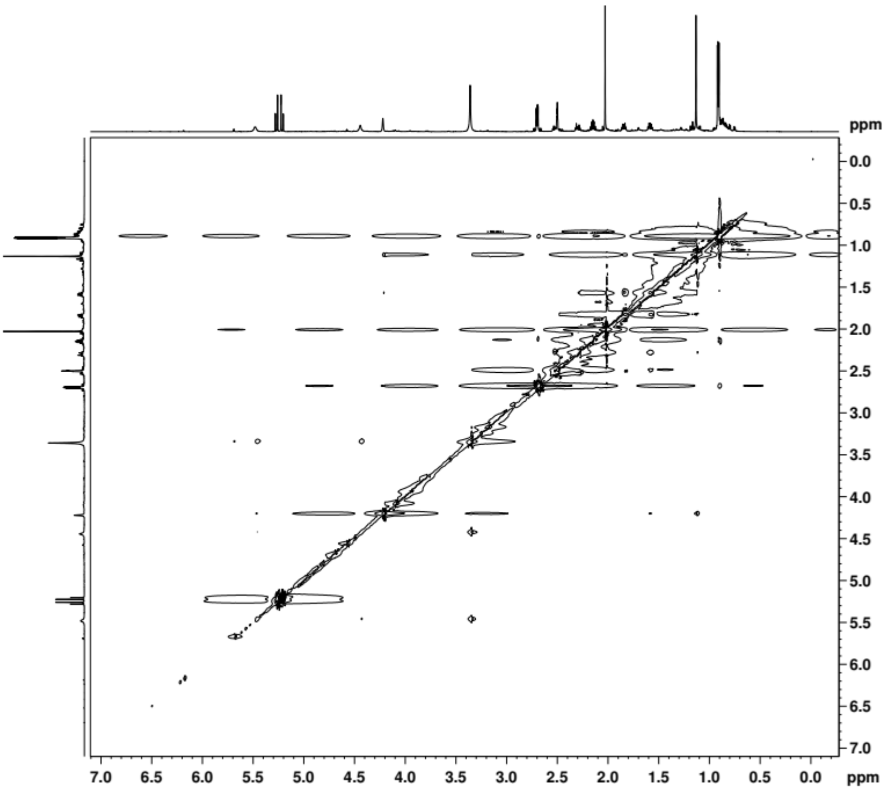

Figure S27. NOESY Spectrum of **4** in DMSO-*d*6.

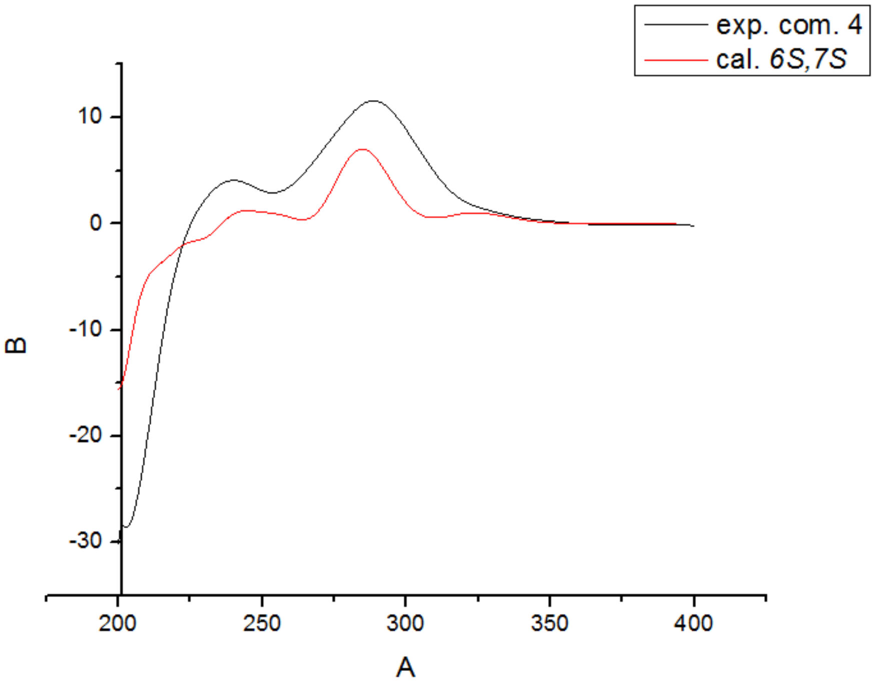

Figure S28. ECD Spectrum of **4.**

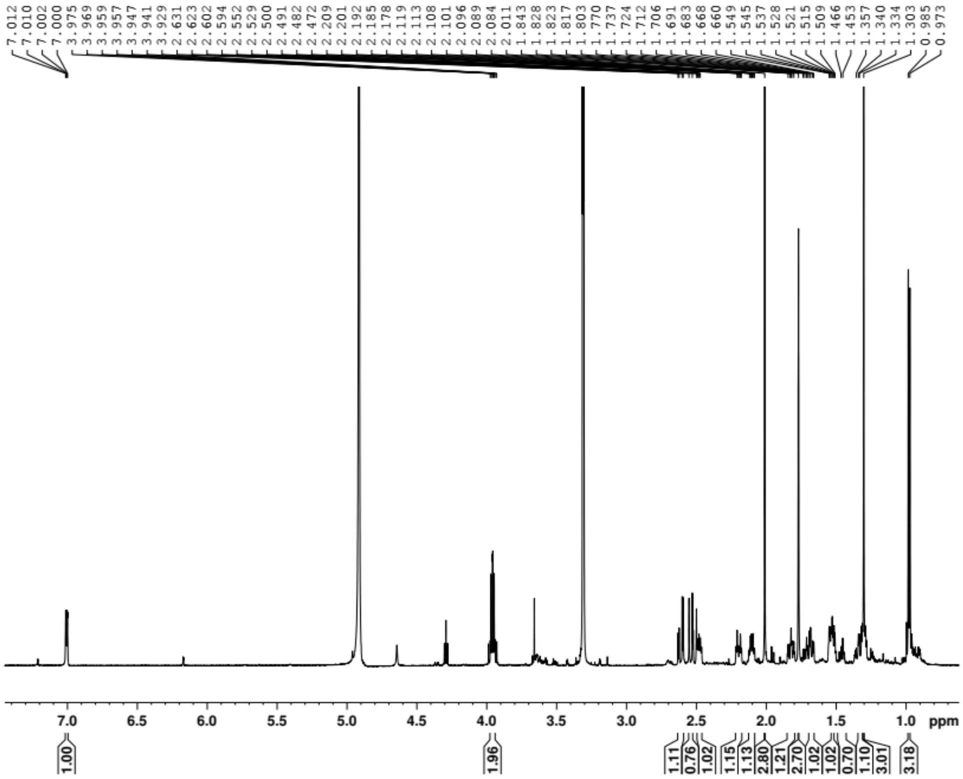

Figure S29. ^{1}H NMR Spectrum of **5** in CD3OD (600 MHz).

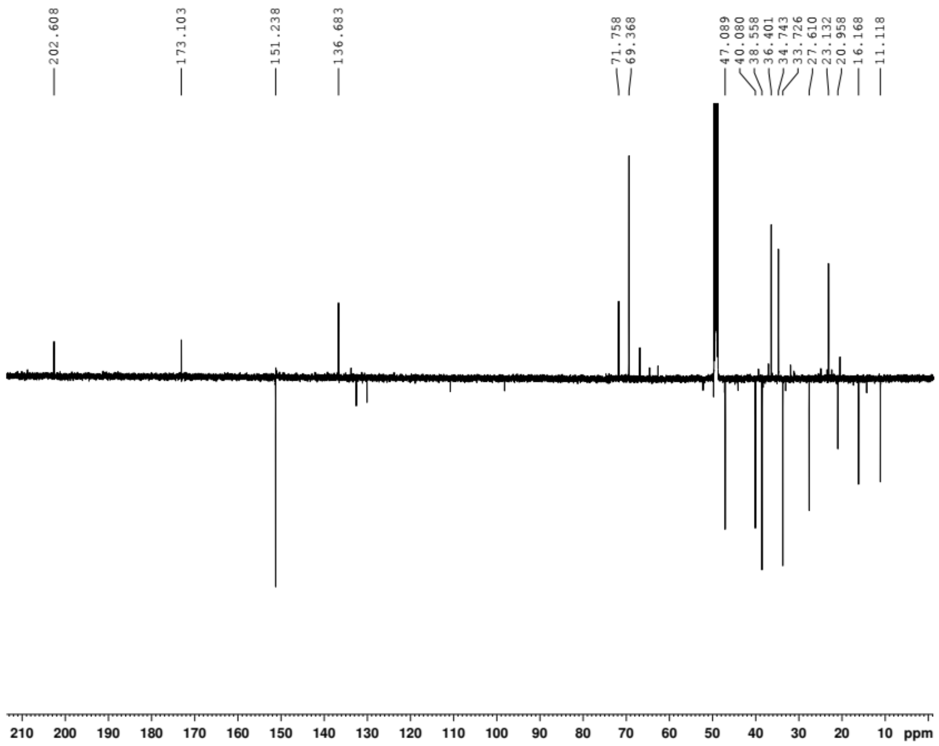

Figure S30. ^{13}C NMR Spectrum of **5** in CD_3OD (150 MHz).

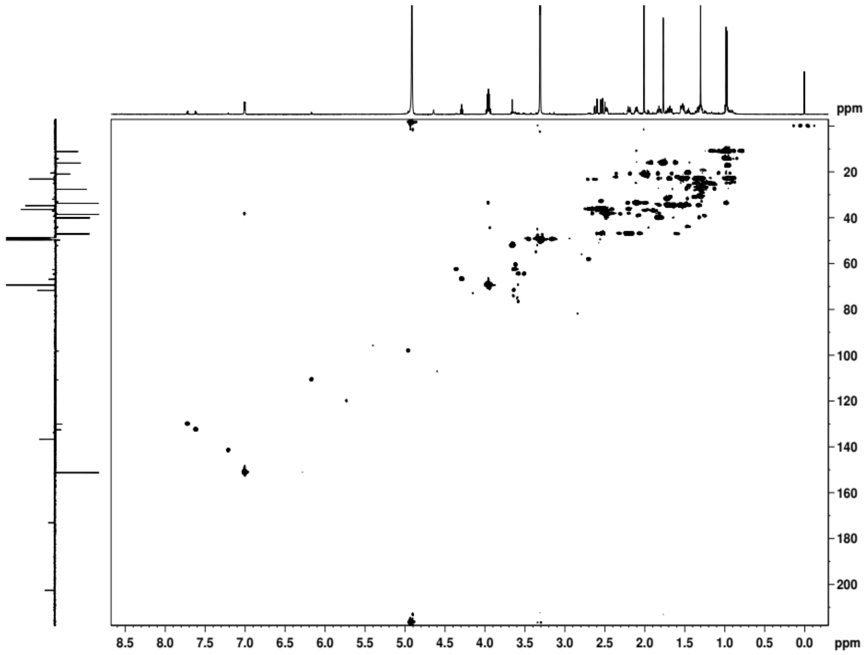

Figure S31. HSQC Spectrum of **5** in CD_3OD.

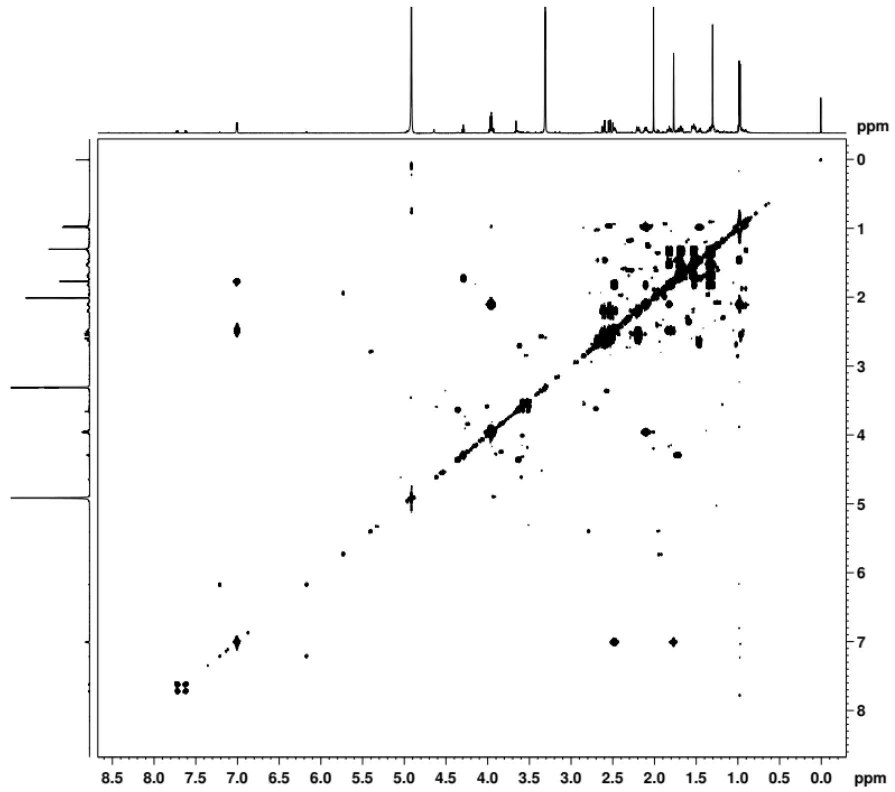

Figure S32. ^{1}H-^{1}H COSY Spectrum of **5** in CD_3OD.

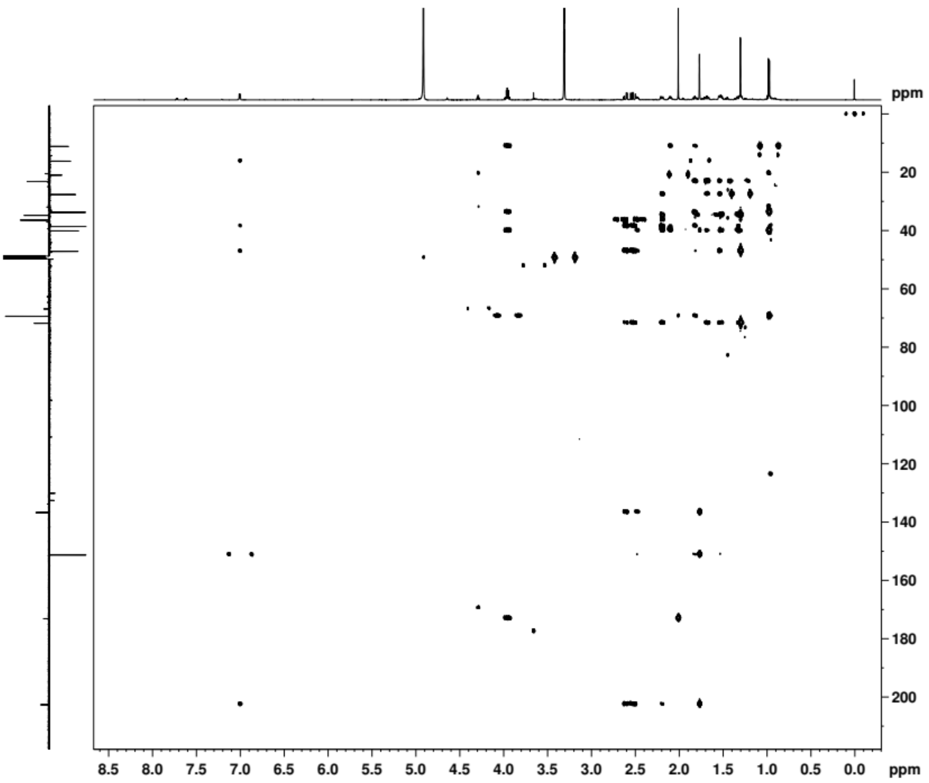

Figure S33. HMBC Spectrum of **5** in CD_3OD.

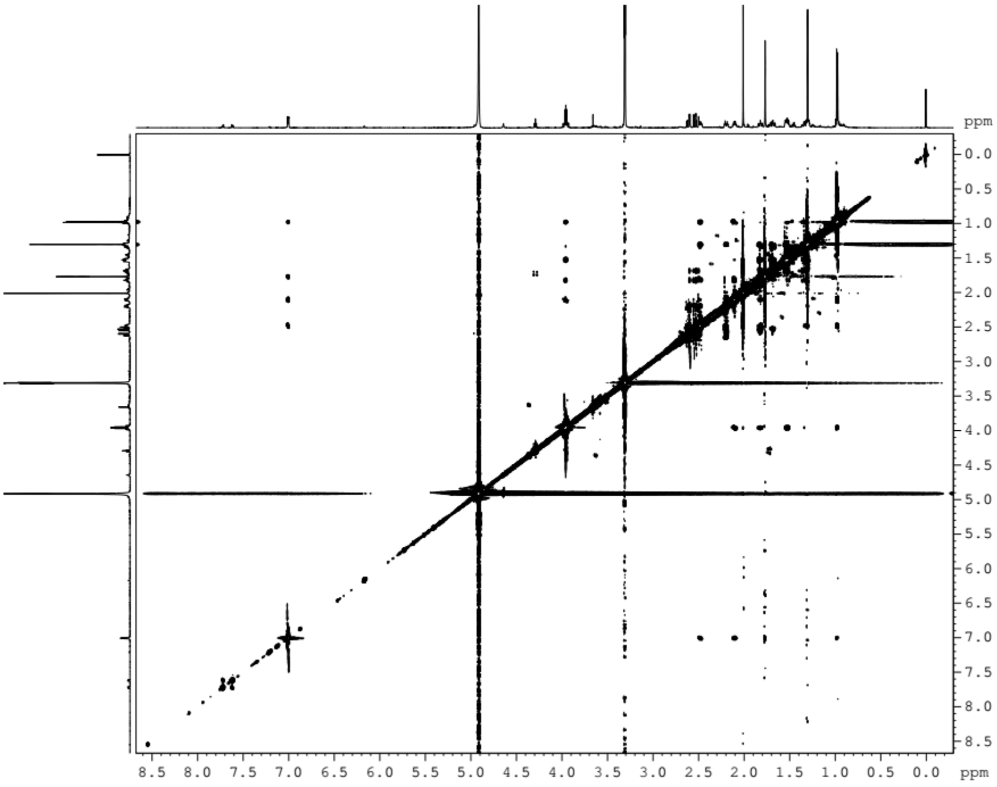

Figure S34. NOESY Spectrum of **5** in CD_3OD.

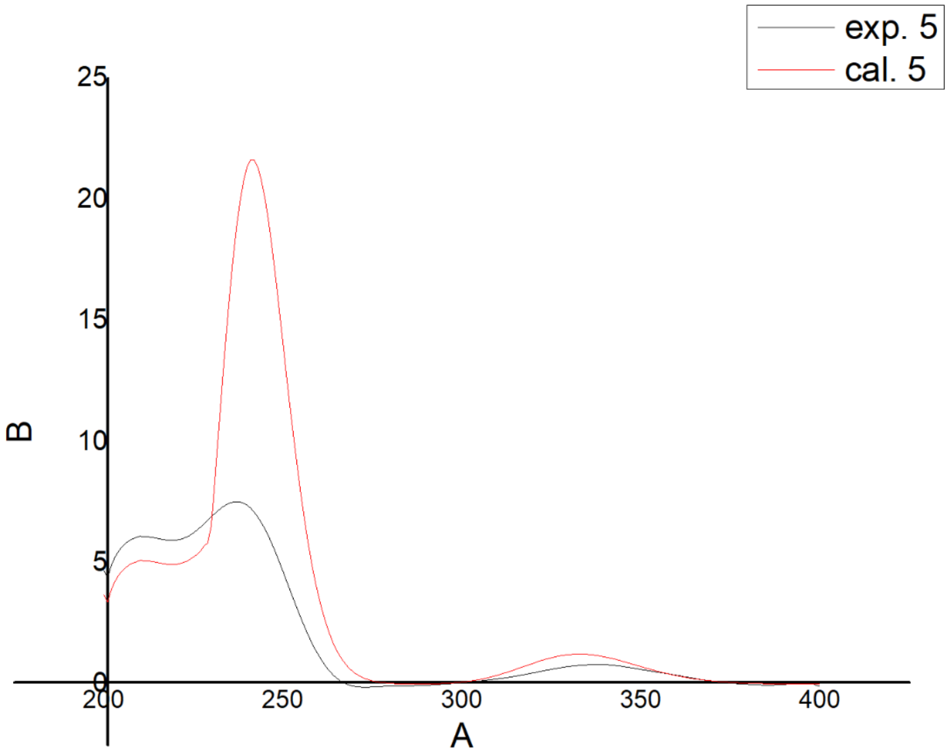

Figure S35. ECD Spectrum of **5**.

Details for ECD calculations of **1**

In general, conformational analyses were carried out via random searching in the Sybyl-X 2.0 using the MMFF94S force field with an energy cutoff of 2.5 kcal/mol.[1] The results showed one lowest energy conformer for both compounds. Subsequently, the conformers were re-optimized using DFT at the PBE0-D3(BJ)/def2-SVP level in MeOH using the polarizable conductor calculation model by the Gaussian 09 program.[2,3] The energies, oscillator strengths, and rotational strengths (velocity) of the first 60 electronic excitations were calculated using the TDDFT methodology at the PBE0/def2-TZVP level in MeOH. The ECD spectra were simulated by the overlapping Gaussian function (half the bandwidth at 1/e peak height, sigma = 0.30 for all).[4] To get the final spectra, the simulated spectra of the conformers were averaged according to the Boltzmann distribution theory and their relative Gibbs free energy *(*ΔG*) at 298.15K.* By comparing the experiment spectra with the calculated model molecules, the absolute configuration of the only chiral center was determined to be *6S,7R,8S*.

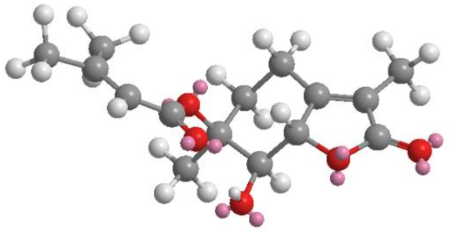

(1**a**)

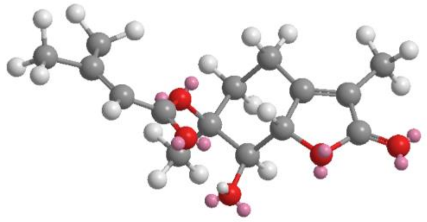

(1**b**)

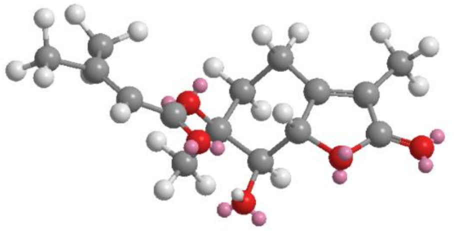

(1**c**)

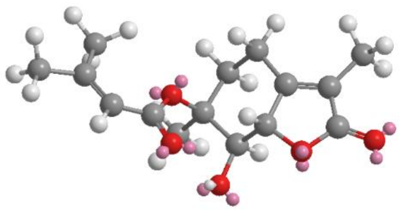

(1**d**)

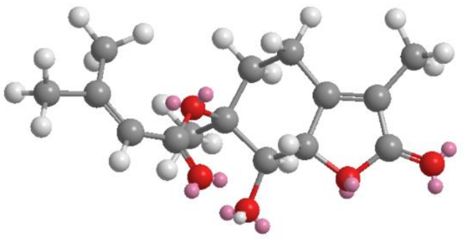

(1**e**)

Figure S36. PBE0/def2-TZVP(methanol) optimized lowest energy conformers for **(*6S,7S,8S*)-1**, (**1a**): optimized conformer 1; (**1b**): optimized conformer 2; (**1c**): optimized conformer 3; (**1d**): optimized conformer 4; (**1e**): optimized conformer 5.

References

1. *Sybyl Software*; Version X 2.0; Tripos Associates Inc.: St. Louis, MO, USA, 2013.
2. Neese, F. The ORCA program system, Wiley Interdiscip. Comput. *Mol. Sci.* **2012**, *2*, 73–78.
3. Neese, F. Software update: the ORCA program system, version 4.0, Wiley Interdiscip. Comput. *Mol. Sci.* **2017**, *8*, e1327.
4. Stephens, P.J.; Harada, N. ECD cotton effect approximated by the Gaussian curve and other methods. *Chirality* **2010**, *22*, 229–233.
